# Prevalence of Sexual Orientation Across 28 Nations and Its Association with Gender Equality, Economic Development, and Individualism

**DOI:** 10.1007/s10508-019-01590-0

**Published:** 2019-12-03

**Authors:** Qazi Rahman, Yin Xu, Richard A. Lippa, Paul L. Vasey

**Affiliations:** 1 Department of Psychology, Institute of Psychiatry, Psychology and Neuroscience, King’s College London, Guy’s Hospital Campus, London, SE1 9RT UK; 2 Department of Psychology, California State University, Fullerton, Fullerton, CA USA; 3 Department of Psychology, University of Lethbridge, Lethbridge, AB Canada

**Keywords:** Sexual orientation, Homosexuality, Culture, Gender roles, Gender equality, Social construction

## Abstract

The prevalence of women’s and men’s heterosexuality, bisexuality, and homosexuality was assessed in 28 nations using data from 191,088 participants from a 2005 BBC Internet survey. Sexual orientation was measured in terms of both self-reported sexual identity and self-reported degree of same-sex attraction. Multilevel modeling analyses revealed that nations’ degrees of gender equality, economic development, and individualism were not significantly associated with men’s or women’s sexual orientation rates across nations. These models controlled for individual-level covariates including age and education level, and nation-level covariates including religion and national sex ratios. Robustness checks included inspecting the confidence intervals for meaningful associations, and further analyses using complete-cases and summary scores of the national indices. These analyses produced the same non-significant results. The relatively stable rates of heterosexuality, bisexuality, and homosexuality observed across nations for both women and men suggest that non-social factors likely may underlie much variation in human sexual orientation. These results do not support frequently offered hypotheses that sexual orientation differences are related to gendered social norms across societies.

**Electronic supplementary material:**

The online version of this article (10.1007/s10508-019-01590-0) contains supplementary material, which is available to authorized users.

## Introduction

Variations in human sexual orientation exist in virtually all modern societies and have been documented in many preindustrial societies as well (Greenberg, 1988; Murray, 2000; Norton, 1997; Whitam & Mathy, 1986). Two empirical facts characterize such variations: (1) heterosexuality is considerably more common than bisexuality or homosexuality, and (2) the percent of men and women who identify as heterosexual, bisexual, or gay/lesbian (in cultures that utilize such categories), and who engage in same-sex sexual interactions, may vary across cultures (Whitam & Mathy, 1986). However, it is not clear whether reports of same-sex attractions (often considered the core psychological component of sexual orientation; Bailey et al., 2016) vary across cultures. An analogy can be made to handedness. Most people (roughly 90%) are right-handed (Coren & Porac, 1977; Frayer et al., 2011; Raymond et al., 1996). However, simultaneously, the proportion of right-handed and non-right-handed individuals varies across cultures, with more non-right-handedness reported in Western than in non-Western cultures (Mandal & Dutta, 2001). These findings have been taken to imply that handedness is largely biologically determined but that cultural pressures can sometimes lead dispositional non-right-handers to conform to social norms of right-handedness, particularly in traditional non-Western nations.

Similarly, we suggest that to the extent that the prevalence of heterosexuality, bisexuality, and homosexuality is consistent across cultures, sexual orientations likely have biological underpinnings (Bailey et al., 2016). At the same time, to the extent that the prevalence of self-reported heterosexuality, bisexuality, and homosexuality varies across cultures (e.g., reported rates of male homosexuality range from about 1.5 to 5%; Gates, 2011; Gómez, Semenyna, Court & Vasey, 2018; Semenyna, Patterson, VanderLaan, & Vasey, 2017; Whitam & Mathy, 1986), such variations may be due, in part, to social and cultural influences. If systematic cross-cultural variation exists in sexual orientation prevalence rates, it may be possible to identify specific cultural factors that are associated with such prevalence rates across cultures. In addition, the influence of such factors may differ for the identity, attraction, and behavioral components of sexual orientation.

A priori, are there candidate factors that might be expected to predict variations in sexual orientation rates across nations? One is gender-related attitudes and the strength of gender roles. In many societies, homosexual identities, attractions, and behaviors constitute violations of gender norms, which typically prescribe normative heterosexuality, participation in heterosexual marriage, and gender-linked activities related to the bearing and rearing of children (Bearman & Bruckner, 2002; Greenberg, 1988; Terry, 1999). This has led some social scientists and scholars in the humanities to argue that sexual orientation is socially influenced by societal gender roles. This is part of a broader argument by such scholars that sexual orientation is a social construct because of the variations in the meaning of same-sex sexuality, its manner of expression, and variations in sexual behavior across cultures and across different historical periods (Bearman & Bruckner, 2002; Fausto-Sterling, 2000; Greenberg, 1988; Risman & Schwartz, 1988; Terry, 1999).

Despite the frequency of such hypotheses, we know of no robust empirical tests, using cross-cultural data, of the hypothesis that sexual orientation is associated with societal gender norms. It is worth noting that these broadly social constructionist perspectives tend not to offer clear predictions about what the relationship between gendered norms and sexual orientation should be. One social constructionist approach argues that homosexuality may be associated with the rise of Western capitalism and middle-class, property-owning family structures (Halperin, 1990; Weeks, 1977). Also taking a social–environmental position, social role theory predicts that sex differences in behavior may be the result of social structures (e.g., greater male than female power or patriarchy), social roles (e.g., economic and domestic division of labor), and gender ideologies that reinforce these patriarchal structures and roles (Eagly, Wood, & Diekman, 2000; Wood & Eagly, 2002). Social role theory therefore predicts that stronger gender roles should be associated with larger sex differences across societies. Gender socialization theories (Ruble & Martin, 1998) argue that the proximate cause of sex differences in behavioral traits are sex-differentiated socialization pressures and practices. These are predicted to generate different behavioral traits in boys and girls, with more gender unequal societies generating larger sex differences than more gender-equal ones (Lippa, 2010).

As a result, taking the perspective of social role and gender socialization theories, we hypothesized that the stronger gender roles and norms were in a given society, the more likely that members of that society would conform to heterosexual identities and sexual interests. Thus, we predicted that gender-egalitarian societies with less rigid gender roles (i.e., those scoring higher on national indicators of gender equality and gender development) would have higher rates of bisexuality and homosexuality than gender-nonegalitarian societies with more rigidly defined gender roles. Because economic development (a potential proxy variable for capitalism) is often positively associated, across nations, with gender-egalitarian attitudes (e.g., Lippa, 2010), we further hypothesized that, across nations, economic development would also be associated with higher rates of bisexual and homosexual identities and attractions.

Individualism–collectivism is another cultural dimension that could be related to the prevalence of heterosexuality, bisexuality, and homosexuality across nations. Cultures high on individualism encourage members to freely express their beliefs, attitudes, and desires and to enact individual identities, even when such identities sometimes violate social norms, whereas cultures high on collectivism strongly encourage members to “fit in” and conform to social norms, roles, and expectations (Hofstede, 1991; Lawrence, 2010; Peabody, 1999). In relation to sexual orientation, we hypothesized that individuals with same-sex sexual attractions in individualistic cultures would be more likely to report and express such attractions and to enact identities consistent with their attractions, whereas individuals with same-sex attractions in collectivist cultures would be more likely not to act on such attractions so as to fit into sexually differentiated cultural roles, and they would be less likely to develop open identities as sexual minorities.

The associations just hypothesized between sociocultural factors (i.e., gender-related attitudes and roles, economic development, and individualism-collectivism) and the prevalence of heterosexuality, bisexuality, and homosexuality across nations might differ for men and women. Baumeister (2000) proposed that women’s sexuality is more “plastic” and influenced by social, cultural, and situational pressures than men’s, whereas men’s sexuality is, in contrast, more biologically channeled than women’s. In a similar vein, Diamond (2008, 2009) described women’s same-sex and other-sex attractions as more variable than men’s, often shifting in response to social settings and relational contexts. Women also report more bisexuality and experience sexual attractions to men and women that are substantially less category-specific in relation to target sex compared to men, which may constitute further evidence of the malleability of female sexual orientation in response to sociocultural forces (Bailey et al., 2016). Thus, we hypothesized that links between social factors and the prevalence of heterosexuality, bisexuality, and homosexuality across nations might be stronger for women than for men.

Finally, we hypothesized that associations between various sociocultural factors and sexual orientation might differ when sexual orientation was assessed in terms of sexual identity (how individuals label themselves—e.g., as “heterosexual,” “bisexual,” “gay,” or “lesbian”) versus when sexual orientation was assessed in terms of sexual attraction (e.g., the degree to which individuals experience and report sexual desire to members of their own sex). Specifically, we hypothesized that sociocultural factors might be associated more strongly with sexual identities than with sexual attraction, because the boundaries of sexual identities are, to some extent, socially defined and may shift depending on cultural pressures more than internal sexual desires do. For example, in some cultures, some men who engage in sex with males nonetheless identify themselves as heterosexual or “straight” (Petterson, Dixson, Little, & Vasey, 2016; Whitam, 1992).

## Method

### Participants

From February through May 2005, the British Broadcasting Corporation (BBC) conducted an Internet survey on human sex differences for use in its documentary, *Secrets of the Sexes*. A total of 255,114 people responded to at least some items in each of the six sections of the survey. A number of published studies have used the BBC data to investigate cross-cultural variations in various traits, and the results of these studies have been consistent with other cross-cultural studies (Lippa, 2009, 2010; Lippa, Collaer, & Peters, 2010).

A number of methodological features of the BBC survey encouraged participants to respond honestly. The survey was taken online and was anonymous, and participants were informed that their data would be used in bona fide academic research. Except for a few questions (such as those inquiring about gender and age, which were used to channel participants to or away from subsequent questions), most questions could be left unanswered by participants who chose not to respond. The BBC survey was long and had multiple sections and, in the current analyses, we used data only from those participants who completed the entire survey. This excluded from the analysis the majority of casual participants who wanted to see “what the survey was like” without necessarily providing conscientious responses. The final question of the BBC survey asked, “Have you answered the questions on this site honestly?” Only one percent of participants responded “no” to this question. The survey language was English. A total of 462,859 participants completed demographic information (Section 1 of the survey) and 255,116 participants completed the entire survey (55.11%) which include sexual orientation items (Reimers, 2007). Only participants who were 18 years of age or older were able to respond to the sexuality-related questions in the survey. It was not possible to know how many people simply clicked on or opened the survey and did not progress any further.

To generate reasonably stable estimates of the prevalence of heterosexuality, bisexuality, and homosexuality in various nations, we restricted our analyses to 28 nations that yielded at least 150 men and 150 women who responded to sexual identity questions: Australia, Austria, Belgium, Bulgaria, Canada, Denmark, Finland, France, Germany, Greece, India, Ireland, Italy, Japan, Malaysia, the Netherlands, New Zealand, Norway, the Philippines, Poland, Romania, Singapore, Spain, Sweden, Switzerland, Turkey, the UK, and the U.S. A total of 191,088 individuals were included in the analysis (about 75% of the 255,116 participants mentioned above). Samples of men and women ranged in size across the 28 nations, with median sample sizes of 541 men and 397 women.

The majority (68%) of participants in the survey were young adults 18 to 40 years of age (median age = 27). Participants tended to be educated—13.5% reported completing postgraduate or professional school, 35.5% university, 9.3% technical or vocational college, 11.5% other colleges, and 29.2% primary or high school. Participants were relatively affluent, with 37.1% reporting an annual income of 0 to 10,000 British pounds, 28.7% an income of 10,000 to 25,000 British pounds, 25.4% an income of 25,000 to 50,000 British pounds, and 8.8% an income of 50,000 British pounds or greater. Participants reported a variety of occupations: 32.1% were students, 64.9% worked in various occupations, and 3.0 reported being unemployed. Participants had to be able to respond to the survey written in English (for further information about the demographics of the BBC sample, see Reimers, 2007).

### Measures

#### Sexual Orientation

Most participants in the BBC study reported their sex and also responded to three questions related to sexual orientation: “What is your sexual orientation? (Response options: “Heterosexual (straight),” “Homosexual (gay/lesbian),” or “Bisexual.” “How sexually attracted are you to men?” and 3) “How sexually attracted are you to women?” (Participants were asked, in response to these items, to rate their degree of attraction on a 7-point scale that ranged from “1—not at all” to “7—very”). Thus, the BBC items tapped identity and attraction but not behavioral components of sexual orientation.

In our analysis, we used each participant’s ordinal responses to the sexual identity question. For participants’ responses to same-sex attraction items, we used both the continuous response on the seven-point scale, and an ordinal measure computed as being “predominantly not sexually attracted to the same-sex” (scoring “1” and “2” on the same-sex attraction scales), “moderately sexually attracted to the same-sex” (scoring “3,” “4,” or “5” on the same-sex attraction scales), and “predominantly sexually attracted to the same-sex” (scoring “6” or “7” on the same-sex attraction scales).

#### National Indices of Gender Equality, Economic Development, and Individualism Traits

National statistics for gender-related development and gender empowerment were taken from the United Nations 2005 and 2001 Human Development Reports (available at http://hdr.undp.org/en/content/human-development-report-2001 and http://hdr.undp.org/en/content/human-development-report-2005). The UN gender-related development index (termed “gender equality” here) assessed nations’ gender equity on three dimensions: health and longevity, standard of living, and knowledge and education. The UN gender empowerment (termed “gender power” here) measure assessed nations’ gender equity on three power dimensions: power over economic resources, participation in economic decision making, and participation in political decision making. In several cases, when 2005 statistics were not available for given nations, we used the 2001 statistics instead. United Nations gender empowerment statistics were not available for two of the 28 nations studied (France and India). Two indices of economic development were also obtained from the UN Human Development reports above (these figures are given for 2003 in those reports): nations’ per capita GDP income in US dollars and life expectancy in years. National scores for individualism-collectivism were taken from Hofstede (1991), and these scores were missing for three of the 28 nations (Bulgaria, Poland, and Romania).

#### Covariates

Serving as possible control variables (Tables 1, 2), participant age and education level (primary or high school, technical or vocational college, other college, university, and postgraduate) were assessed as individual-level covariates, and national sex ratios and dominant religion (Protestant, Catholic, Eastern Orthodox, Muslim, Buddhist, mixed Christian, and mixed) served as nation-level covariates in multilevel models (see below). National sex ratios for people 15–64 years old were obtained from the World Fact Book 2005, a publication of the United States Central Intelligence Agency (https://www.cia.gov/library/publications/download/download-2005/index.html). Information regarding the dominant religion of a country was obtained from the *US Department of State International Religious Freedom Report 2004* (for more details, see Lippa, 2009).

**Table 1:** National indices (including covariates, sex ratio, and religion) by nation

Country	*N*	Gender equality	Life expectancy	Income (US $)	Sex ratio	Gender power	Individualism-collectivism^a^	Dominant religion
Australia	8003	0.95	80.30	26275	1.02	0.83	90	Mixed Christian
Austria	398	0.93	79.00	31289	1.01	0.78	55	Catholic
Belgium	1322	0.94	78.90	29096	1.02	0.83	75	Catholic
Bulgaria	381	0.81	72.20	2539	0.97	0.60	–	Eastern Orthodox
Canada	11673	0.95	80.00	27079	1.01	0.81	80	Mixed Christian
Denmark	779	0.94	77.20	39332	1.02	0.86	74	Protestant
Finland	1628	0.94	78.50	31058	1.02	0.83	63	Protestant
France	965	0.94	79.50	29410	1.00	–	71	Catholic
Germany	1484	0.93	78.70	29115	1.04	0.81	67	Mixed Christian
Greece	843	0.91	78.30	15608	1.00	0.59	35	Eastern Orthodox
India	3193	0.59	63.30	564	1.07	–	48	Hindu
Ireland	488	0.94	77.70	38487	1.00	0.72	70	Catholic
Italy	443	0.93	80.10	25471	1.02	0.59	76	Catholic
Japan	474	0.94	82.00	33713	1.01	0.53	46	Buddhist
Malaysia	775	0.79	73.20	4187	1.01	0.50	26	Mixed
Netherlands	2108	0.94	78.40	31532	1.03	0.81	80	Mixed Christian
New Zealand	1990	0.93	79.10	19847	1.01	0.77	79	Mixed Christian
Norway	565	0.96	79.40	48412	1.03	0.93	69	Protestant
Philippines	407	0.76	70.40	989	0.99	0.53	32	Catholic
Poland	441	0.86	74.30	5487	0.99	0.61	–	Catholic
Romania	359	0.79	71.30	2619	0.99	0.49	–	Eastern Orthodox
Singapore	1748	0.87	78.70	21492	0.95	0.65	20	Mixed
Spain	817	0.92	79.50	20404	1.01	0.75	51	Catholic
Sweden	1232	0.95	80.20	33676	1.03	0.85	71	Protestant
Switzerland	557	0.95	80.50	43553	1.02	0.80	67	Mixed Christian
Turkey	1294	0.74	68.70	3399	1.03	0.29	37	Muslim
UK	95793	0.94	78.40	30253	1.02	0.72	89	Protestant
USA	50928	0.94	77.40	37648	1.00	0.79	91	Protestant

“–” indicates missing for that nation

^a^Absolute range is 0–100

**Table 2:** Mean age and education level (proportions) by nation

Country	*N*	Age (in years)	Education				
			Primary/high school	Technical college	Other colleges	University	Postgraduate
Australia	8003	30	0.37	0.13	0.06	0.34	0.11
Austria	398	28	0.39	0.10	0.12	0.30	0.09
Belgium	1322	30	0.31	0.06	0.11	0.37	0.14
Bulgaria	381	25	0.25	0.06	0.03	0.58	0.09
Canada	11673	31	0.32	0.12	0.11	0.36	0.10
Denmark	779	30	0.30	0.13	0.18	0.32	0.06
Finland	1628	26	0.41	0.12	0.11	0.31	0.05
France	965	32	0.12	0.05	0.04	0.50	0.29
Germany	1484	29	0.26	0.09	0.10	0.39	0.16
Greece	843	29	0.09	0.09	0.09	0.41	0.32
India	3193	26	0.06	0.13	0.05	0.41	0.35
Ireland	488	27	0.30	0.07	0.12	0.35	0.15
Italy	443	32	0.24	0.06	0.07	0.42	0.21
Japan	474	31	0.11	0.05	0.05	0.59	0.20
Malaysia	775	26	0.18	0.03	0.15	0.51	0.13
Netherlands	2108	30	0.30	0.11	0.14	0.36	0.09
New Zealand	1990	33	0.30	0.14	0.06	0.37	0.13
Norway	565	27	0.34	0.11	0.12	0.34	0.09
Philippines	407	26	0.16	0.03	0.10	0.60	0.10
Poland	441	26	0.35	0.05	0.07	0.43	0.10
Romania	359	25	0.26	0.03	0.03	0.53	0.15
Singapore	1748	23	0.33	0.10	0.18	0.33	0.06
Spain	817	31	0.21	0.08	0.07	0.51	0.14
Sweden	1232	28	0.32	0.08	0.10	0.42	0.08
Switzerland	557	33	0.16	0.08	0.09	0.41	0.26
Turkey	1294	29	0.08	0.01	0.02	0.65	0.23
UK	95793	29	0.30	0.10	0.12	0.34	0.13
USA	50928	31	0.28	0.07	0.13	0.37	0.14

In addition, we computed men’s and women’s mean age and education levels for each national sample. Education was assessed in terms of the percent of men and women who reported completing at least a high school education in each nation. These measures proved to be highly correlated with gender equality, economic development, and individualism-collectivism, across nations. When correlations were computed between the four demographic factors (men’s mean age, women’s mean age, men’s education, women’s education) and the five overlapping national indices (gender equality, gender power, income, life expectancy, and individualism–collectivism), 18 of 20 correlations were significant, with a median correlation of .56. In general, higher mean ages and higher education levels in both males and females were associated with greater gender equality, economic development, and individualism. Analysis of the demographic factors showed that male and female samples, across nations, tended to be well matched. The correlation between men’s and women’s mean age, across nations, was *r*(28) = .94, *p* < .001, and the corresponding correlation for education level was *r*(28) = .93, *p* < .001.

### Statistical Analysis

#### Missing Data

The variables included in the current study had 0.002–16.81% missing information within the analysis sample. These missing data were handled by a technique called multiple imputation which is useful for large datasets such as ours. It quantifies uncertainty about the missing data by creating different imputed data sets and combining results obtained from them (Sterne et al., 2009). This increases power and overcomes some possible biases as incomplete data is included in analyses. Multiple imputation was used because the commonly used complete-cases analysis approach causes a substantial loss of precision and power (Sterne et al., 2009). Complete-cases analysis may also cause bias when data are missing at random instead of missing completely at random (Sterne et al., 2009).

In our data, there were at least 359 individuals within each cluster (nation) and 28 clusters. Statisticians generally recommend that missing data be imputed separately within each cluster (Graham, 2009). Individual-level missing variables were imputed separately within each cluster using individual-level and observed cluster-level variables (Gelman & Hill, 2007). Then the cluster-level missing variables were imputed using the cluster-level variables and aggregated forms (national means) of the individual-level variables (Gelman & Hill, 2007).

Prior to imputation, we examined, using logistic regression, whether the observed variables predicted “missingness.” For the imputation model, recommendations for multilevel studies suggest that all variables in the analysis model should be included (White, Royston, & Wood, 2011). Thus, the outcome variable (sexual orientation), predictors (national indices), and covariates were included. In addition, it is recommended that the number of imputations is at least as large as the percentage of missing data (White et al., 2011). Thus, we used 17 imputations. We used the chained equations algorithm (MICE) model since we had a combination of continuous and categorical variables. We used *predictive mean matching* for continuous variables since this approach makes no distributional assumption. Trace plots and other diagnostics indicated no cause for concern regarding the imputed values. The main multilevel models (see below) were based on the imputed data. For completeness, we compare those results with those based on complete-cases (see Supplemental tables).

#### Principal Component Analysis for Intercorrelations Between National Indices

We expected national indices to be highly intercorrelated. However, it would not be theoretically meaningful to compute a single composite measure of all these variables. Since gender power and gender equality were highly correlated, *r*(26) = .84, *p* < .01, and life expectancy and income were also highly correlated, *r*(28) = .79, *p* < .01, summary scores incorporating these indicators were constructed to generate two more meaningful composites: gender development and economic development. We applied principal component analysis and used the loadings on the first principal component as item weightings to generate a summary score for gender development and economic development separately. The first component explained 91.93% and 89.27% of the variation gender development and economic development, respectively. We compared the results for multilevel models using the individual national indices and those using these summary measures.

#### Multilevel Models

Because participants (Level 1 units) were nested within nations (Level 2 units), there were dependencies in the data. Therefore, we used a multilevel modeling approach to the main research question (to test whether national indices of gender equality, economic development, and individualism predict sexual orientation). Data were analyzed in STATA version 15 using multilevel models with random intercepts and fixed slopes (since the nation-level variables did not vary across individuals within a country). Given the variation in age and education level between participants, and in sex ratio and dominant religion between nations, we used age and education as Level 1 covariates and sex ratio and religion as Level 2 covariates in the models. Sexual identity was treated as an ordinal outcome variable, and same-sex attractions as both ordinal and continuous outcome variables. Analyses were stratified by sex. Results show the odds ratios and 95% confidence intervals for ordinal outcome variable, and regression coefficients (*beta*) and 95% confidence intervals for continuous outcome. The model also provided the variance partition coefficient which refers to the proportion of unexplained variance (after accounting for predictors and covariates in the model) in the outcome due to differences between nations.

## Results

### Descriptive Statistics and Sex Differences for Prevalence of Sexual Orientation

Table 3 shows the breakdown of sexual orientation for each nation. Table 4 shows the mean percent of men and women, across nations, who self-identified as heterosexual, bisexual, and gay/lesbian and the mean percent of men and women who reported being predominantly not attracted to their own sex, moderately attracted to their own sex, and predominantly attracted to their own sex. The percent of men who reported a heterosexual identity (90.0%) was significantly larger than the percent who reported being predominantly not attracted to men (82.6%), paired data *t*(27) = 14.81, *p* < .001. Similarly, the percent of women who reported a heterosexual identity (90.7%) was significantly larger than the percent who reported being predominantly not attracted to women (66.2%), paired-data *t*(27) = 25.70, *p* < .001. Thus, men and women who labeled themselves as “heterosexual” included a substantial number of individuals who also reported being moderately or predominantly attracted to their own sex.

**Table 3:** Sexual attraction and sexual identity (proportions) by nation

Country	*N*	Sexual attraction (categorical)			Sexual identity		
		Predominantly not attracted to the same-sex	Moderately attracted to the same-sex	Predominantly attracted to the same-sex	Straight	Bisexual	Gay/Lesbian
Australia	8003	0.74	0.19	0.07	0.90	0.06	0.04
Austria	398	0.67	0.26	0.07	0.88	0.09	0.03
Belgium	1322	0.78	0.16	0.05	0.91	0.06	0.04
Bulgaria	381	0.74	0.20	0.06	0.93	0.06	0.02
Canada	11673	0.75	0.19	0.07	0.90	0.06	0.04
Denmark	779	0.77	0.18	0.06	0.93	0.04	0.03
Finland	1628	0.71	0.22	0.07	0.86	0.11	0.03
France	965	0.74	0.17	0.09	0.87	0.07	0.06
Germany	1484	0.71	0.21	0.08	0.87	0.08	0.05
Greece	843	0.84	0.12	0.04	0.94	0.04	0.03
India	3193	0.89	0.08	0.03	0.93	0.06	0.01
Ireland	488	0.81	0.12	0.07	0.91	0.05	0.03
Italy	443	0.75	0.17	0.08	0.87	0.08	0.05
Japan	474	0.72	0.20	0.07	0.92	0.05	0.04
Malaysia	775	0.72	0.20	0.08	0.90	0.06	0.04
Netherlands	2108	0.70	0.19	0.10	0.85	0.07	0.08
New Zealand	1990	0.74	0.20	0.06	0.90	0.06	0.04
Norway	565	0.80	0.15	0.05	0.92	0.06	0.02
Philippines	407	0.69	0.20	0.11	0.86	0.07	0.07
Poland	441	0.70	0.21	0.09	0.91	0.06	0.03
Romania	359	0.81	0.14	0.05	0.94	0.04	0.03
Singapore	1748	0.72	0.22	0.06	0.90	0.06	0.04
Spain	817	0.73	0.19	0.08	0.91	0.04	0.06
Sweden	1232	0.79	0.16	0.05	0.92	0.06	0.02
Switzerland	557	0.80	0.14	0.06	0.91	0.06	0.03
Turkey	1294	0.80	0.14	0.06	0.96	0.03	0.01
UK	95793	0.78	0.15	0.06	0.91	0.05	0.04
USA	50928	0.73	0.18	0.08	0.88	0.07	0.05

For sexual attraction, the original score which is measured on a 7-point scale was transformed into 3 groups: “predominantly not sexually attracted to the same-sex” (scoring “1” and “2” on the same-sex attraction scales), “moderately sexually attracted to the same-sex” (scoring “3,” “4,” or “5” on the same-sex attraction scales), and “predominantly sexually attracted to the same-sex” (scoring “6” or “7” on the same-sex attraction scales)

**Table 4:** Means and SDs of men’s and women’s sexual orientation prevalence rates across 28 nations

Sexual orientation	Men	Women
Sexual identity		
Heterosexual	90.0% (3.58)	90.7% (3.58)
Bisexual	5.1% (1.75)	7.2% (2.94)
Gay/Lesbian	4.9% (2.57)	2.1% (1.17)
Sexual attraction		
Predominantly not attracted to the same sex	82.6% (4.48)	66.2% (6.85)
Moderately attracted to the same sex	10.2% (2.91)	27.3% (5.67)
Predominantly attracted to the same sex	7.2% (2.87)	6.5% (1.91)

For sexual attraction, the original score which is measured on a 7-point scale was transformed into 3 groups: “predominantly not sexually attracted to the same-sex” (scoring “1” and “2” on the same-sex attraction scales), “moderately sexually attracted to the same-sex” (scoring “3,” “4,” or “5” on the same-sex attraction scales), and “predominantly sexually attracted to the same-sex” (scoring “6” or “7” on the same-sex attraction scales)

Across nations, mean rates of male and female heterosexual identity (90.0% and 90.7%) did not differ, independent *t*(54) = − .72. However, a higher percent of men (4.9%) than women (2.1%) reported a homosexual identity, *t*(54) = 5.22, *p* < .001, whereas a lower percent of men (5.1%) than women (7.2%) reported a bisexual identity, *t*(54) = − 3.25, *p* = .002.

There was no consistent evidence, across nations, for sex differences in the variability of sexual orientation prevalence rates. Levene’s test for the equality of variances showed no significant differences in the variance of prevalence rates for men’s and women’s self-reported heterosexual or bisexual identities across nations. However, prevalence rates of men’s homosexual identity were more variable, across nations, than prevalence rates of women’s lesbian identity, *p* = .009. When sexual orientation was assessed in terms of participants’ same-sex attraction, women were more variable than men, across nations, in their prevalence of being moderately attracted to the same sex, *p* < .004. However, women and men did not differ significantly in the variability of their prevalence of not being attracted to the same sex and being predominantly attracted to the same sex (all *p*s > .05).

Finally, the relatively small cross-nation *SD*s reported in Table 4—particularly for prevalence rates for homosexual identities and predominant same-sex attractions—suggest substantial consistency across nations. Thus, despite the existence of variations across nations, sexual orientation rates were nonetheless relatively stable across nations.

### Associations Between National Indices and Sexual Orientation for Men and Women

Tables 5 and 6 show the results of multilevel models using imputed data for men and women, respectively. There were no significant associations between national indices of gender equality, gender power, life expectancy, national income, and individualism scores, on the one hand, and sexual identity or sexual attraction measures, on the other hand. For men, the significant odds ratio for the association between gender power and sexual identity (directionally opposite to predictions) had a confidence interval containing 1. Therefore, the evidence was inconclusive that this was a meaningfully significant association. The variation partition coefficients were small, indicating that the prevalence of sexual orientation was quite stable across nations. As robustness checks, we conducted further models using complete-cases and summary scores. Models using complete-cases also did not change the results much (note that in the few cases that odds ratios were significant, confidence intervals included zero or 1 depending on the statistic), and neither did models using the summary scores derived from the PCA for complete-cases and imputed data (see Supplementary tables).

**Table 5:** Multilevel model results for men after multiple imputation

National indices	Sexual identity	Sexual attraction (ordinal)	Sexual attraction (continuous)
Gender equality	1.05 (0.92, 1.18)	1.07 (0.96, 1.20)	0.03 (–0.04, 0.10)
Gender power	0.98* (0.96, 1.00)	0.99 (0.97, 1.01)	–0.01 (–0.02, 0.01)
Life expectancy	0.92 (0.79, 1.07)	0.91 (0.8, 1.04)	–0.05 (–0.14, 0.04)
Income	1.00 (1.00, 1.00)	1.00 (1.00, 1.00)	–0.00 (–0.00, 0.00)
Individualism-collectivism	1.00 (0.98, 1.01)	0.99 (0.98, 1.00)	–0.01 (–0.01, 0.00)
Variance partition coefficient	0.55%	0.40%	0.26%

All models were adjusted for age and education level as individual-level covariates and religion and sex ratio as nation-level covariates. For sexual identity, heterosexual men are the reference group. For sexual attraction (ordinal), men who are predominantly not sexually attracted to the same-sex are the reference group. We reported adjusted odds ratios and 95% confidence intervals for ordinal outcome variable, and regression coefficients (*beta*) and 95% confidence intervals for continuous outcome. The variance partition coefficient is interpreted as the proportion of the total residual variance in the propensity to be nonheterosexual/attracted to the same-sex is due to differences between nations

*p < .05

**Table 6:** Multilevel model results for women after multiple imputation

National indices	Sexual identity	Sexual attraction (ordinal)	Sexual attraction (continuous)
Gender equality	0.99 (0.85, 1.16)	0.98 (0.88, 1.10)	–0.01 (–0.11, 0.08)
Gender power	1.00 (0.98, 1.03)	1.01 (0.99, 1.03)	0.01 (–0.00, 0.03)
Life expectancy	0.99 (0.81, 1.21)	1.00 (0.88, 1.15)	0.01 (–0.10, 0.12)
Income	1.00 (1.00, 1.00)	1.00 (1.00, 1.00)	–0.00 (–0.00, 0.00)
Individualism-collectivism	1.00 (0.98, 1.01)	1.00 (0.99, 1.01)	–0.00 (–0.01, 0.01)
Variance partition coefficient	1.13%	0.54%	0.55%

All models were adjusted for age and education level as individual-level covariates and religion and sex ratio as nation-level covariates. For sexual identity, heterosexual women are the reference group. For sexual attraction (ordinal), women who are predominantly not sexually attracted to the same-sex are the reference group. We reported adjusted odds ratios and 95% confidence intervals for ordinal outcome variable, and regression coefficients (*beta*) and 95% confidence intervals for a continuous outcome. The variance partition coefficient is interpreted as the proportion of the total residual variance in the propensity to be nonheterosexual/attracted to the same-sex is due to differences between nations

As an additional robustness check of the non-significant findings, we conducted a power calculation. The large number of individuals in the sample suggests that statistical power to detect significant associations (odds ratios or regression coefficients) was large. However, estimating statistical power for multilevel models is complex and involves using sample sizes at both levels and estimates of the variance components (Scherbaum & Ferreter, 2009). For ease of interpretation, here we illustrate the power of the study using the continuous outcome of sexual attractions. Using guidelines provided by Scherbaum and Ferreter (2009) at the 5% level, a sample size of 28 nations and 150 individuals within each nation was sufficient for a detection of small effect size with 90% power. We were thus able to detect a small association with low probability of a false negative.

## Discussion

The central question addressed by the current research was: Are national factors such as gender equality, economic development, and individualism-collectivism related to the national prevalence of various sexual orientations, across 28 nations? Our analyses also tested the frequently offered hypothesis that sexual orientation rates may be associated with gender norms and social roles (Bearman & Bruckner, 2002; Greenberg, 1988; Terry, 1999). The use of a large international dataset allowed us to test whether countries that differed in gender egalitarianism and rigidity of gender roles (as indexed by national indicators of gender equality and gender empowerment) also differed in the prevalence of various sexual orientations. We found no compelling evidence that this was the case. While the present results were not significant, they demonstrate that several theoretically important predictor variables (national levels of gender equality, economic development, and individualism) were not much associated with important outcome variables (sexual identity and same-sex attractions) in a very large sample with sufficient statistical power. The non-significant results were also inconsistent with the notion that women’s sexual identities and same-sex and other-sex attractions are more linked to cultural and social factors than men’s were (Bailey et al., 2016; Baumeister, 2000). Furthermore, there was no evidence that national indices were more strongly related to identity than to attraction-based measures of sexual orientation. Finally, the pattern of associations did not seem to result from the fact that prevalence rates were more variable, in general, for women than men across nations. Indeed, when assessed in terms of sexual identity, prevalence rates for male homosexual identity were more variable than prevalence rates for lesbian identity were.

Some factors that may be related to the prevalence of men’s sexual orientation were not assessed in the current study. One candidate supported by previous research is participants’ average number of older brothers in a given national sample (and the correlated factor of the average size of participants’ family of rearing in a given national sample). Many studies have shown that the more older biological brothers a man has, the more likely he is to be gay (Blanchard, 2018). This “fraternal birth order effect” is thought to result from biological processes—each additional male fetus carried by a woman increases the likelihood of maternal immunological reactions against male factors in fetal tissue, and these immunological reactions then influence the development of subsequent male fetuses (Bogaert et al., 2018). A prediction that follows from the fraternal birth order effect is that nations with larger mean family sizes at the time of participants’ births should, on average, have higher rates of male but not female homosexuality among adult probands (Bogaert, 2004). Although not tested in the current study, this hypothesis suggests the possibility that biological as well social factors could be associated with the prevalence of heterosexuality, bisexuality, and homosexuality, across nations, and furthermore that associations with biological as well as social factors may sometimes differ for men and women.

The current study had several limitations. One pertains to the sexual identity categories used. In some cultures, one’s degree of sexual attraction to men and women is simply not a basis upon which individuals construct identities. Cultural variations in the construal of same-sex and other-sex attractions have also been affected by our use of an English language survey. While other cultures may sometime use sexual identity terms that are comparable to those employed in Western countries, such terms may have different meanings across cultures, as for example when a man identifies as “straight,” but nonetheless engages in sexual activity with same-sex partners (e.g., Petterson et al., 2016). In some cultures (e.g., those with “third gender” categories), sexual orientation might be seen as a basis for identity, but at the same time, some or all of the Western terms that are commonly used to denote sexual orientation may not be employed (e.g., Asthana & Oostvogels, 2001; Petterson et al., 2016). Similar issues can even characterize some subcultures within Western nations, in which asking members whether they are “heterosexual,” “homosexual,” or “bisexual” is discouraged (e.g., Denizet-Lewis, 2010). In the context of the current study, it is worth noting that all participants, in fact, identified themselves using one of the provided sexual identity terms, and thus they seemed willing to use the categories of “heterosexual,” “bisexual,” and “homosexual” as a basis for self-classification.

A second limitation is that the national samples in the BBC survey were not random or representative. Thus, each national subsample is not necessarily representative of national patterns overall. As the participants in all countries come from a sample of BBC consumers, there may be cross-national homogeneity built into the sampling frame. As noted earlier, participants tended to be young, affluent, and educated (as well as able to understand the English language). Compared to other cross-cultural studies on college student samples, the BBC data included data from non-college populations who came from various locations within the various countries and who varied in age and various demographic characteristics.

One obvious direction for future research is to replicate the current findings with data from representative samples of men and women from diverse nations. Many of the nations studied in the current study were European with a number of notable exceptions (e.g., India, Japan, Malaysia, Philippines, Singapore, Turkey). The unequal sample sizes across nations (some nations contained more people than others) is unlikely to bias the estimation of the parameters of interest. One of the advantages of using multilevel models is their tolerance of unequal samples and other unbalanced data structures. Simulation studies suggest that group-level sample size is somewhat more important than total sample size, and large individual-level sample sizes can compensate for small numbers of groups (for review, see Maas & Hox, 2005). Naturally, any estimates of grand means (e.g., across all nations) will be more weighted toward countries with larger sample sizes which is why researchers should use multilevel models when nesting is inherent in the study design.

It is also important to note that the concept of national culture (insofar as that is captured by UN indices) has been questioned by scholars in personality and social psychology. While the concept of national cultures is disputed, other research suggests there may be between-nation differences in average personality traits and that some of these may correlate with sociopolitical structures (e.g., having democratic institutions; Barceló, 2017; Schmitt, Allik, McCrae, & Benet-Martínez 2007). In this context, Hofstede’s measures of individualism and collectivism have also been criticized. As cultures (especially those in closer geographic proximity) tend to become more similar (perhaps due to economic factors such as globalization), it is possible that consistency in psychological traits across cultures may also be driven by globalized sexual norms. While the analysis presented here accounts for the statistical dependencies introduced by these issues, the findings are specific to the BBC sample examined. Social attitudes toward sexual orientation may also have changed since the BBC survey was taken. Thus, further tests of these questions will be needed in other, more representative and recent cross-cultural datasets.

The use of multilevel models allowed us to use nation-level data to draw inferences at the individual level. In other words, it allowed us to test the potential influence of national gender equality on individuals’ sexual identity and desire. However, the relationship between variables could theoretically be different at other levels of analysis. For example, societal or structural-level gender egalitarianism could influence intermediate proximate mechanisms, such as parental gender socialization or internalization of gender stereotypes (or other gender norms), which then influence the development of sexual orientation differences. However, the effects of factors such as parental socialization on sexual orientation appear to be weak based on existing research evidence (Bailey et al., 2016). Furthermore, many country-level variables may be clustered in world regions (e.g., Europe, North America). While multilevel model can accommodate such effects (e.g., by simply adding another data level in a hierarchical model), it is unlikely that levels of gender egalitarianism differ sufficiently between countries within a world region (e.g., between all European countries) for us to detect such associations with sufficient statistical power.

Finally, it is worth noting that although the national samples of men and women studied in the BBC survey were not representative of their larger national populations in some ways, the male and female samples were nonetheless well matched on demographic factors such as age and education levels. Thus, the apparent absence of sex differences in the current study—e.g., there appeared to be no difference between men and women in the relation between sociocultural factors and sexual orientation—was present despite the fact that male and female samples were matched on key factors.

In conclusion, our analyses did not yield a significant association between national indicators of gender equality, economic development, and individualism-collectivism traits and identity-based or desire-based measures of sexual orientation across 28 countries in men and women. This provides new evidence that questions the power of factors such as gendered norms, gender roles, and gender socialization to account for variations in the prevalence of sexual orientations across nations. Future empirical studies are needed to better test the extent to which national gender norms and economic factors are related to variations in the expression of sexual orientation across nations.

## Electronic supplementary material

Below is the link to the electronic supplementary material.

Supplementary material 1 (DOCX 18 kb)
