# Supplementary material for: Prevalence of Sexual Orientation Across 28 Nations and Its Association with Gender Equality, Economic Development, and Individualism
Source: Arch Sex Behav. 2019 Dec 3;49(2):595–606. doi: 10.1007/s10508-019-01590-0 (PMC7031179; doi:10.1007/s10508-019-01590-0)
Supplement: Supplementary file 1 — Supplementary material 1 (DOCX 18 kb) [file 10508_2019_1590_MOESM1_ESM.docx]

**Supplemental Table 1** Multilevel analysis for men using complete-cases

| National indices | Sexual identity | Sexual attraction  (ordinal) | Sexual attraction  (continuous) |
| --- | --- | --- | --- |
| Gender equality | 1.06 (0.93, 1.21) | 1.09 (0.97, 1.22) | 0.05 (-0.03, 0.12) |
| Gender power | 0.98* (0.96, 1.00) | 0.99 (0.97, 1.01) | -0.01 (-0.02, 0.01) |
| Life expectancy | 0.88 (0.76, 1.02) | 0.89 (0.78, 1.01) | -0.07 (-0.15, 0.01) |
| Income | 1.00 (1.00, 1.00) | 1.00 (1.00, 1.00) | 0.00 (0.00, 0.00) |
| Individualism-collectivism | 1.00 (0.98, 1.01) | 0.99 (0.98,1.00) | -0.01 (-0.01, 0.00) |

*Note*. All models were adjusted for age and education-level as individual-level covariates, and religion and sex ratio as nation-level covariates. For sexual identity, heterosexual men are the reference group. For sexual attraction (ordinal), men who are predominantly not sexually attracted to the same-sex are the reference group. We reported adjusted odds ratios and 95% confidence intervals for ordinal outcome variable, and regression coefficients (*beta*) and 95% confidence intervals for continuous outcome.

**p* < .05.

**Supplemental Table 2** Multilevel analysis for women using complete-cases

| National indices | Sexual identity | Sexual attraction  (ordinal) | Sexual attraction  (continuous) |
| --- | --- | --- | --- |
| Gender equality | 1.16 (0.98, 1.38) | 1.09 (0.98, 1.22) | 0.07 (-0.02, 0.16) |
| Gender power | 1.00 (0.97, 1.03) | 1.00 (0.98, 1.02) | 0.00 (-0.01, 0.02) |
| Life expectancy | 0.84 (0.69, 1.02) | 0.90 (0.80, 1.01) | -0.07 (-0.17, 0.03) |
| Income | 1.00 (1.00, 1.00) | 1.00* (1.00, 1.00) | -0.00 (-0.00, -0.00) |
| Individualism-collectivism | 0.98* (0.97, 1.00) | 0.99* (0.98, 1.00) | -0.01* (-0.02, -0.00) |

*Note*. All models were adjusted for age and education-level as individual-level covariates, and religion and sex ratio as nation-level covariates. For sexual identity, heterosexual women are the reference group. For sexual attraction (ordinal), women who are predominantly not sexually attracted to the same-sex are the reference group. We reported adjusted odds ratios and 95% confidence intervals for ordinal outcome variable, and regression coefficients (*beta*) and 95% confidence intervals for continuous outcome.

**p* < .05.

**Supplemental Table 3** Multilevel analysis for men using complete-cases and summary scores

| National indices | Sexual identity | Sexual attraction  (ordinal) | Sexual attraction  (continuous) |
| --- | --- | --- | --- |
| Gender development | 0.97** (0.95, 0.99) | 0.99 (0.98, 1.01) | -0.01 (-0.02, 0.01) |
| Economic development | 1.00 (1.00, 1.00) | 1.00 (1.00, 1.00) | -0.00 (-0.00, 0.00) |
| Individualism-collectivism | 1.00 (0.99, 1.01) | 1.00(0.99, 1.00) | -0.00 (-0.01, 0.00) |

*Note*. All models were adjusted for age and education-level as individual-level covariates, and religion and sex ratio as nation-level covariates. For sexual identity, heterosexual men are the reference group. For sexual attraction (ordinal), men who are predominantly not sexually attracted to the same-sex are the reference group. We reported adjusted odds ratios and 95% confidence intervals for ordinal outcome variable, and regression coefficients (*beta*) and 95% confidence intervals for continuous outcome.

***p* < .01.

**Supplemental Table 4** Multilevel analysis for women using complete-cases and summary scores

| National indices | Sexual identity | Sexual attraction  (ordinal) | Sexual attraction  (continuous) |
| --- | --- | --- | --- |
| Gender development | 1.01 (0.98, 1.04) | 1.01 (1.00, 1.03) | 0.01 (0.00, 0.02) |
| Economic development | 1.00 (1.00, 1.00) | 1.00 (1.00, 1.00) | -0.00 (-0.00, 0.00) |
| Individualism-collectivism | 0.99 (0.98, 1.00) | 0.99 (0.99, 1.00) | -0.00 (-0.01, 0.00) |

*Note*. All models were adjusted for age and education-level as individual-level covariates, and religion and sex ratio as nation-level covariates. For sexual identity, heterosexual women are the reference group. For sexual attraction (ordinal), women who are predominantly not sexually attracted to the same-sex are the reference group. We reported adjusted odds ratios and 95% confidence intervals for ordinal outcome variable, and regression coefficients (*beta*) and 95% confidence intervals for continuous outcome.

**Supplemental Table 5** Multilevel analysis for men using summary scores after multiple imputation

| National indices | Sexual identity | Sexual attraction  (ordinal) | Sexual attraction  (continuous) |
| --- | --- | --- | --- |
| Gender development | 0.97** (0.96, 0.99) | 1.00 (0.98, 1.01) | -0.00 (-0.02, 0.01) |
| Economic development | 1.00 (1.00, 1.00) | 1.00 (1.00, 1.00) | -0.00 (-0.00, 0.00) |
| Individualism-collectivism | 1.00 (0.99, 1.01) | 1.00 (0.99, 1.00) | 0.00 (-0.01, 0.00) |

*Note*. All models were adjusted for age and education-level as individual-level covariates, and religion and sex ratio as nation-level covariates. For sexual identity, heterosexual men are the reference group. For sexual attraction (ordinal), men who are predominantly not sexually attracted to the same-sex are the reference group. We reported adjusted odds ratios and 95% confidence intervals for ordinal outcome variable, and regression coefficients (*beta*) and 95% confidence intervals for continuous outcome.

***p* < .01

**Supplemental Table 6** Multilevel analysis for women using summary scores after multiple imputation

| National indices | Sexual identity | Sexual attraction  (ordinal) | Sexual attraction  (continuous) |
| --- | --- | --- | --- |
| Gender development | 1.00 (0.98, 1.02) | 1.01 (0.99, 1.03) | 0.01 (-0.00, 0.02) |
| Economic development | 1.00 (1.00, 1.00) | 1.00 (1.00, 1.00) | -0.00 (-0.00, 0.00) |
| Individualism-collectivism | 0.99 (0.98, 1.01) | 1.00 (0.99, 1.00) | -0.00 (-0.01, 0.00) |

*Note*. All models were adjusted for age and education-level as individual-level covariates, and religion and sex ratio as nation-level covariates. For sexual identity, heterosexual women are the reference group. For sexual attraction (ordinal), women who are predominantly not sexually attracted to the same-sex are the reference group. We reported adjusted odds ratios and 95% confidence intervals for ordinal outcome variable, and regression coefficients (*beta*) and 95% confidence intervals for continuous outcome.
